# Sand-Frictions in Cutaneous Diseases

**Published:** 1877-03

**Authors:** 


Sand-Frictions in Cutaneous Diseases.—Under the above title, Dr. Ellinger, of Stuttgart (*Wien. Med. Woch.*, No. 45), describes a practice he has followed with great success in certain diseases of the skin. Having occasion to seek for some additional remedy in treating two cases of acne of the face in young persons, he employed for this purpose white sand, and in the course of ten or twelve days the skin became clean and smooth, and has so continued since. Recommending the practice to others, he received such confirmation of its good effects that he gave it a trial in various other slight but obstinate cutaneous affections. In ephelis he found the effects longer in being produced, but very satisfactory; and in a case of ptyriasis versicolor the scales disappeared in a few days, the skin losing all its roughness a week later. In a case of acne rosacea affecting the chin and cheeks of a girl, after six weeks' frictions the eruptions disappeared, leaving the skin smooth and white. In other cases of psoriasis palmaris, lichen albus, and prurigo, occurring in young persons, the sand proved equally efficacious.

The selection of sand to be employed is of importance, for while large granules are obviously unsuitable, yet a too large proportion of mere dust renders the frictions of no avail. It should be so sifted as to separate for use granules the size of a half or whole poppy-seed. Two kinds may indeed be prepared: a finer for the tender skin of the face, and a coarser for the trunk and extremities. In eruptions of the face, the skin, before bedtime, should first be washed with soap and water, and allowed to remain moist. In about half an hour after, some moistened sand should be rubbed into the affected parts for a short time, according to the tolerance and the necessity of the case. Any portion of the sand that adheres should be washed off with a sponge, or, better still, brushed off. In eruptions that only occupy a limited portion of trunk or limbs, wherever it is possible, a Priesnitz compress should be first applied to the parts, and the sand rubbed in when they have thus become softened; they being afterwards cleansed by a sponge or brush. When large portions of the body are occupied by the eruption, baths prolonged for one or two hours should precede washing with soap and the application of the sand.

With all his patients Dr. Ellinger has made no change in their mode of life, and has abstained from administering medicine, whether internally or externally. For those who prefer everything to smell of the apothecary, he has sometimes added a little oil of thyme to the sand; and of late he has used a paste made of sand, carbonate of potash (one part to five), and water, which seems to have some advantage over the simple sand. He strongly recommends this also to clinical teachers and prosectors for cleansing the hands. He also thinks sand-friction of importance as a means of keeping the skin in a healthy state, a fact, indeed, that may be demonstrated microscopically by comparing the water of a bath in which sand has been used with that of one in which it has not been employed. The frictions are, too, especially indicated in severe internal chronic diseases, with relaxed, inactive skin—seborrhœa tabescentum; and they might probably be advantageously substituted, using coarse sand, for the severe procedures recommended lately by Dr. Hebra, Jr., in the treatment of lupus and other ulcers.

